# Benzodiazepines, Z-drugs and the risk of hip fracture: A systematic review and meta-analysis

**DOI:** 10.1371/journal.pone.0174730

**Published:** 2017-04-27

**Authors:** Karen Donnelly, Robert Bracchi, Jonathan Hewitt, Philip A. Routledge, Ben Carter

**Affiliations:** 1 Pharmacology, Therapeutics and Toxicology, Cardiff University School of Medicine, Academic Centre, University Hospital Llandough, Cardiff, United Kingdom; 2 Institute of Primary Care and Public Health, Cardiff University, School of Medicine, Neuadd Meirionnydd, Cardiff, United Kingdom; 3 Department of Biostatistics and Health Informatics, Institute of Psychiatry, Psychology and Neuroscience, King’s College London, London, United Kingdom; 4 Cochrane Skin Group, School of Medicine, The University of Nottingham, Nottingham, United Kingdom; Garvan Institute of Medical Research, AUSTRALIA

## Abstract

**Background:**

Hip fractures in the older person lead to an increased risk of mortality, poorer quality of life and increased morbidity. Benzodiazepine (BNZ) use is associated with increased hip fracture rate, consequently Z-drugs are fast becoming the physician’s hypnotic prescription of choice yet data on their use is limited. We compared the risk of hip fracture associated with Z-drugs and BNZ medications, respectively, and examined if this risk varied with longer-term use.

**Methods and findings:**

We carried out a systematic review of the literature and meta-analysis. MEDLINE and SCOPUS were searched to identify studies involving BNZ or Z-drugs and the risk of hip fracture up to May 2015. Each included study was quality-assessed. A pooled relative risk of hip fracture was calculated using the generic inverse variance method, with a random effects model, with the length of hypnotic usage as a subgroup. Both BNZ, and Z-drug use respectively, were significantly associated with an increased risk of hip fracture (RR = 1.52, 95% CI 1.37–1.68; and RR = 1.90, 95% CI 1.68–2.13). Short-term use of BNZ and Z-drugs respectively, was also associated with the greatest risk of hip fracture (RR = 2.40, 95% CI 1.88–3.05 and RR = 2.39, 95% CI 1.74–3.29).

**Conclusions:**

There is strong evidence that both BNZ and Z-drugs are associated with an increased risk of hip fracture in the older person, and there is little difference between their respective risks. Patients newly prescribed these medicines are at the greatest risk of hip fracture. Clinicians and policy makers need to consider the increased risk of fallings and hip fracture particularly amongst new users of these medications.

## Introduction

Hip fractures are associated with repeat fractures and substantial morbidity and mortality. It has been reported that one third of older people die within the year following hip fracture [1, 2]. Many who survive face significantly reduced capacity to carry out activities of daily living and one third require adjustment to institutionalised living [3]. Post-hip fracture, one year all-cause-mortality has remained constant in the UK from 2000 to 2010, with an increased risk of 3.5 for males and 2.4 for females [4]. In the US, one year all-cause mortality is estimated at 24% [5].

The Center for Disease Control estimated that at least 300,000 older people are hospitalised in the US per annum due to hip fracture [6] In Europe the total number of hip fractures is predicted to reach 4.5 million by the year 2025 [7]. Hip fracture carries a major economic burden. Sahota and co-workers reported median cost per UK hip fracture as £9,429 [€10,896] for care home residents and £14,435 [€16,681] for those requiring transition post-hip fracture into a care home [8]. The annual costs of hip fracture are approximately £2 billion in the UK [2] and $10.3–15.2 billion in the US respectively [5, 9, 10].

Indeed there is a well-established association between psychotropic medications and falls [11, 12]. Benzodiazepines (BNZ) have long been associated with fracture risk [13–16]. The national institute for clinical excellence (NICE) reported that an estimated 10–30% of chronic users are BNZ dependent with evidence of tolerance or drug seeking behaviour and that 50% of all users experience symptoms of withdrawal [17]. BNZ are subdivided according to their elimination time (half-life): short (t_½_ = 1–12 hours), intermediate (t_½_ = 12 to 40 hours) or long (t_½_ = 40–250 hours)[18].

Zolpidem, zaleplon and zopiclone are classed as non-benzodiazepine hypnotics (often referred to as a group as “Z-drugs"). Z-drugs were designed with a shorter elimination time (t½ = 1–7 hours) to act as a more clinically attractive alternative to BNZ [19]. They aim to achieve hypnotic effect without the undesired side effects of BNZ, such as dependence or sedation on the following day. Nevertheless, the pharmacological action of Z-drugs are similar to BNZ as it involved the benzodiazepine receptor site which are associated with gamma-aminobutyric acid (GABA) receptors. Siriwardena et al. 2006 reported that general practitioners believed Z-drugs are safer and more effective than BNZ [20]. In 2014, it was estimated that 26.1% of the adult UK population have ever taken a BNZ or Z-drug[21]. In 2004 and 2008, the annual prevalence of BNZ use has been estimated at 4% of the Canadian population and 5.2% of the US population respectively [22]. In Europe, interventions amongst general practitioners have been successful in decreasing BNZ prescriptions [23]. Despite this, concerns have been reported that prescriptions of Z-drugs such as zopiclone have increased over the past decade [22, 24–26].

The current literature base illustrates that there is an increased risk of hip fracture following BNZ use [27, 28] and zolpidem use [29] respectively. Furthermore, NICE has warned that there is a lack of compelling evidence to distinguish between the safety of short-acting BNZ and Z-drugs[17]. However, importantly clinical perceptions differ [20] and there is debate surrounding the suitable length of hypnotic prescription. In the United States, the Compendium of Therapeutic Choices (CTC) has recommended a short course of hypnotics combined with good sleep hygiene, for the treatment of insomnia [30]. In Canada, the Physicians’ Desk Reference has recommended short term BNZ prescription only (two to four weeks) [31–34]. In the UK the British National Formulary (BNF) has recommended that BNZ should be used to treat insomnia “only when it is severe, disabling, or causing the patient extreme distress” [35]. The BNF also states that “The use of benzodiazepines to treat short-term ‘mild’ anxiety is inappropriate”, and that they are indicated for the short-term relief (two to four weeks only) of anxiety that is severe, disabling, or causing the patient unacceptable distress, occurring alone or in association with insomnia or short-term psychosomatic, organic, or psychotic illness[35]. However others, have suggested significant balance impairment can occur following a single dose [36] and Wagner *et* al. determined BNZ use to be most dangerous within the first 15 days compared continuous use (RR = 1.74 1.07–2.82) [37].

The objectives of this study were to investigate the association between (1) BNZ use and hip fracture risk and (2) Z-drug use and hip fracture risk. We aimed to study this relationship according to length of use.

## Methods

### Inclusion and exclusion criteria

This study followed the PRISMA checklist and flowchart [38] (S1 File). A protocol was developed and agreed prior to the review (S2 File). Studies were included if all of the following criteria applied (i) designed as a randomised controlled trial, cohort or case-control study (ii) reported outcome was hip fracture (ICD-10: S72) or fragility fracture (within which outcome ≥70% of fractures were hip fractures) (iii) included patients were prescribed either BNZ or Z-drug, or were matched as a non-exposed control population (iv) the study population were aged at least 50 years old and with a mean age over 65. Clinically, clonazepam is frequently used as an anti-epileptic medication rather than as a hypnotic and were therefore excluded [39]

### Search strategy

Two databases (i) Medline via Ovid (S3 File) (EMBASE, Ovid, Psych INFO) and (ii) Scopus were systematically searched on 11th May, 2015. Searches were independently carried out by two reviewers (KD, BC), and disagreements were resolved by discussion. The search was limited to studies published in the English language.

### Definition of exposure

Exposure was categorised into two main subgroups: exposure to BNZ v non-exposure; and exposure to Z-drugs v non-exposure. BNZ exposure was defined as patients prescribed diazepam, lorazepam, chlordiazepoxide, oxazepam, temazepam, nitrazepam, loprazolam or clobazam [40]. Z-drug exposure was defined as those prescribed zaleplon, zolpidem or zopiclone [35]. Length of usage was defined from the first prescription date, provided there was at least one hypnotic free month. Short term use was defined as those prescribed medication for up to 14 days, medium term use 15 days to 30 days, and long term use was longer than one month, mixed use was a combination of medium and long-term users.

### Study selection & quality assessment

Database results were reviewed by examining titles and abstracts. Full articles were examined for methodological quality using the Newcastle-Ottawa quality assessment tool. Studies were assessed to be of good, fair or poor quality [41]. Quality assessment was carried out independently by two reviewers (KD and BC), disagreements were resolved by discussion.

### Data extraction and characteristics of included studies

The outcome was the proportion of participants that had a hip fracture (ICD-10:S72). The characteristics of the eligible studies included: author, year, location, environment, study design, age profile, length of exposure, length of follow up, total participants and adjustment for confounders with particular attention to dosage. Data was separately extracted by two reviewers (KD, BC) and discrepancies were resolved following discussion.

### Data synthesis

The risk of hip fracture in those exposed to a BNZ (or Z-drugs), compared to patients not taking these medications. The measure of effect was the adjusted relative risk (RR) with the associated 95% confidence interval (95% CI). Included comparisons were studies of: people using BNZ compared to those not exposed; and people using Z-drugs compared to those not exposure. Non-randomised study designs were described narratively and only pooled into a meta-analysis if their context, population, medication (including delivery) were considered clinically similar [42]. A pooled relative risk using generic inverse variance methods with a random effects model was used in RevMan 5.3 [43, 44]. The shortest time point was included in the pooled analysis from studies that reported at multiple time-points [37, 45–47]. Funnel plots to explore reporting bias were examined for all pooled meta-analyses, or subgroup of greater than ten studies.

#### Subgroups analysis and assessment for heterogeneity

Statistical heterogeneity was summarised using an *I*^2^ statistic. Where *I*^2^ was reported higher than 75%, subgroups were explored to explain the heterogeneity [43]. Subgroups used to explore heterogeneity were: length of use; case mix of patients (insomniac-only studies or not); and type of study design (population based or non-population based studies).

## Results

**Fig 1:**
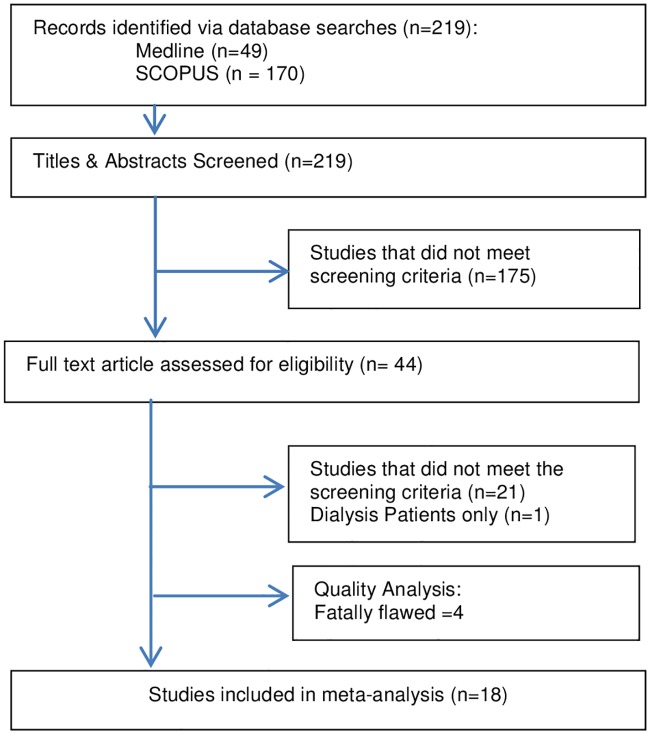
PRISMA flowchart: Study selection for systematic review and meta analysis.

There were 219 studies identified. After screening of abstracts and titles, 44 articles met the inclusion criteria which led to 22 being quality assessed and 18 included studies (Fig 1).

### Study characteristics & quality assessment

The included studies were published between 1995 and 11th of May 2015. No randomised controlled trials were identified; twelve case-control studies and 10 cohort studies were identified. Four studies were excluded on the basis of quality [two fair studies [48, 49] and two poor studies [50, 51]]. Overall eighteen studies were included; nine case control studies [14–16, 46, 52–56] and nine cohort studies [37, 45, 47, 57–62]. Further details can be found in Table 1. Clinical differences for definition of drug exposure were compared across all studies. Studies were compared for differences in the context of their setting including: of location, design, fracture type, mean age, sample size, length of drug exposure and adjustment for confounders with particular attention to dose. The included sample sizes ranged from 500 to 906,422 participants. The mean age of participants in the included studies ranged from 72.0 to 84.3 years. Further details can be found in S4 File.

**Table 1:** Quality assessment using the Newcastle Ottawa scale.

	Selection (S)				Comparability (C)		Exposure /Outcome E/O			Sub Total assessment			
	1	2	3	4	1a	1b	1	2	3	S^+^	C^&^	E/O^&^	Conclusion
**Case Control Studies:**													
(Berry et al. 2013)	*	*	*	*	*	*	*	**No**	*	Good	Good	Good	Good
(Chang et al. 2008)	*	*	*	*	*	*	*	*	*	Good	Good	Good	Good
(Coutinho et al. 2008)	*	*	*	*	*	*	**No**	*	*	Good	Good	Good	Good
(Golden et al. 2010)	*	*	*	*	*	*	*	*	*	Good	Good	Good	Good
(Hoffmann et al. 2006)	**No**	*	**No**	*	*	*	*	**No**	*	**Fair**	**Good**	**Good**	**Fair**
(Jensen et al. 1991)	*	*	*	*	*	*	**No**	**Unclear**	**No**	**Good**	**Good**	**Poor**	**Poor**
(Kang et al. 2012)	*	*	*	*	*	*	*	**No**	*	Good	Good	Good	Good
(Lichtenstein et al. 1994)	*	*	*	**No**	*	*	*	**No**	**No**	**Good**	**Good**	**Poor**	**Poor**
(Perreault et al. 2008)	*	*	*	*	*	*	*	*	*	Good	Good	Good	Good
(Pierfitte et al. 2001)	*	*	*	*	*	*	*	*	*	Good	Good	Good	Good
(Wang et al. 2001)	*	*	*	*	*	*	*	*	*	Good	Good	Good	Good
(Zint et al. 2010)	*	*	**No**	*	*	*	*	*	*	Good	Good	Good	Good
	**Selection (S)**				**Comparability (C)**		**Outcome (O)**						
	**1**	**2**	**3**	**4**	**1a**	**1b**	**1**	**2**	**3**				
**Cohort Studies:**													
(Bakken et al. 2014)	*	*	*	*	*	*	*	*	*	Good	Good	Good	Good
(Chan et al. 2010)	*	*	*	**No**	*	*	*	*	*	Good	Good	Good	Good
(Cummings et al. 1995)	*	*	*	*	*	*	*	*	*	Good	Good	Good	Good
(Ensrud et al. 2003)	*	*	*	*	*	*	*	*	*	Good	Good	Good	Good
(Finkle et. al 2011)	*	*	*	*	*	*	*	*	*	Good	Good	Good	Good
(Guo et al. 1998)	*	*	*	*	*	*	*	*	*	Good	Good	Good	Good
(Huybrechts et al. 2011)	**No**	**No**	*	*	*	*	*	*	**No**	**Fair**	**Good**	**Good**	**Fair**
(Kragh et al. 2011)	*	*	*	**No**	*	*	*	*	*	Good	Good	Good	Good
(Thorell et al. 2014)	*	*	*	**No**	*	*	*	*	*	Good	Good	Good	Good
(Wagner et al. 2004)	*	*	*	**No**	*	*	*	*	*	Good	Good	Good	Good

^+^Domain scored: 0–1 (Poor); 2 (Fair); 3+ (Good);

^&^Domain scored: 0 (Poor); 1 (Fair); 2+ (Good).

* Domain acceptable

### Effect of BNZ compared to non-exposure

**Fig 2:**
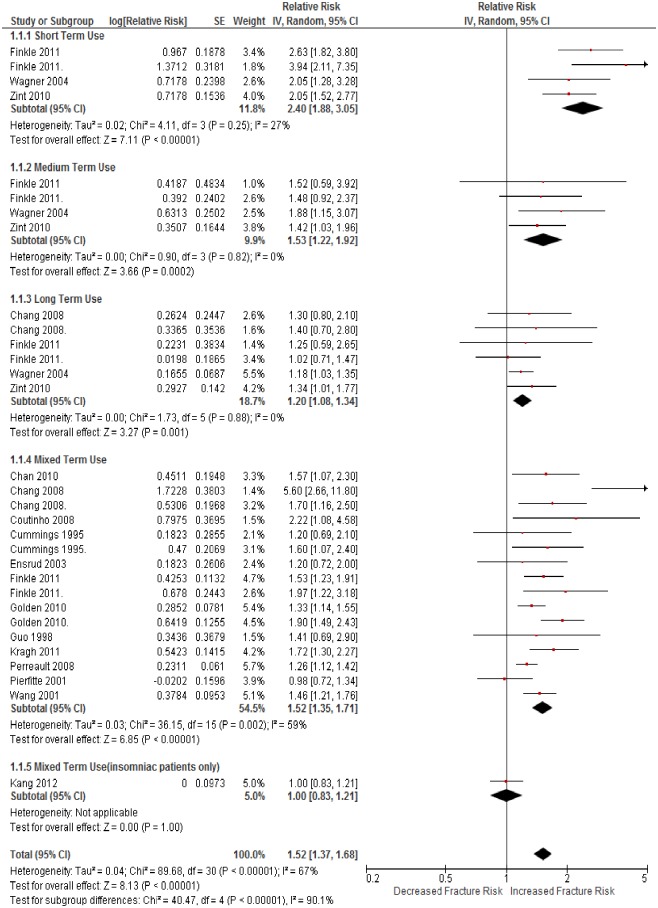
The adjusted relative risk of hip fracture in participants who used BNZ, compared to people who did not, by the length of use.

Eighteen studies were included [13–16, 37, 45, 47, 52–62]. There was an associated increase in hip fracture risk with BNZ use (RR = 1.52, 95% CI 1.37–1.68, P<0.001, *I*^2^ = 67%; Fig 2). Severe heterogeneity was explained by the varying length of usage; therefore the risk of fracture was dependent on the length of use.

Short term use carried a 140% increased risk of hip fracture (RR = 2.40, 95% CI 1.88–3.05, P<0.001, *I*^2^ = 27%). Medium term use carried 53% increased risk (RR = 1.53, 95% CI 1.22–1.92, P<0.001, *I*^2^ = 0%) and long term use carried 20% increased risk (RR = 1.20, 95% CI 1.08–1.34, P<0.001, *I*^2^ = 0%). The mixed length of use subgroup carried a 52% increased risk (RR = 1.52, 95% CI 1.35–1.71, P<0.001, *I*^2^ = 59%), but given the heterogeneous nature of this group, this finding should be interpreted cautiously. We explored possible reporting bias within and between subgroup using funnel plots, and conclude there was no reason to suspect this bias after accounting for the length of use excluding mixed use studies (S5 File).

### Population based studies

**Fig 3:**
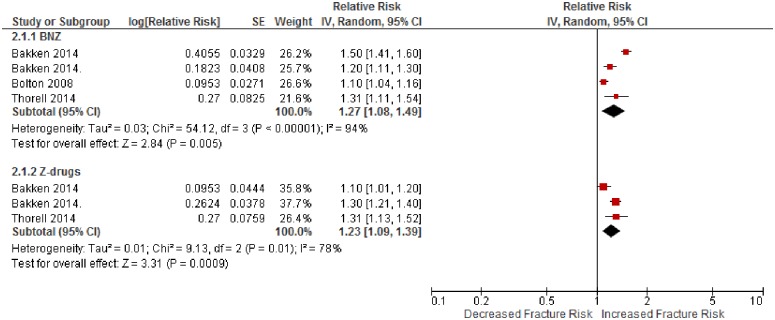
The adjusted relative risk of hip fracture in population-based studies of participants who used BNZ or a Z-drug, compared to people who did not.

Population based studies were presented separately, with a range of results [13, 45, 62] (Fig 3). Severe heterogeneity was exhibited, due to their clinical diversity and magnitude of effect. Thus, no pooling was carried out. However, all population based studies demonstrated an increased risk of hip fracture following BNZ and following Z-drugs [13, 45, 62].

### Effect of Z-drugs compared to non-exposure

**Fig 4:**
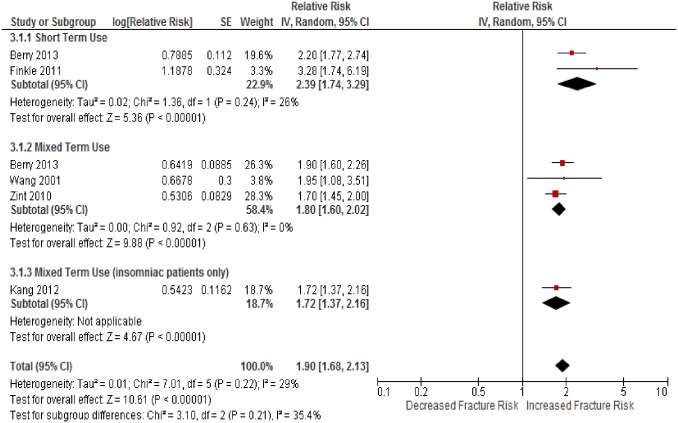
The adjusted relative risk of hip fracture in participants who used a Z-drug, compared to people who did not, by the length of use.

There were 6 studies included [46, 47, 52, 55, 56, 62]. There was an associated increase in hip fracture risk with Z-drug use (RR = 1.90, 95% CI 1.68–2.13, P<0.001, *I*^2^ = 26%) (Fig 4). Short term use carried a 139% increased risk of hip fracture (RR = 2.39, 95% CI 1.74–3.29, P<0.001, *I*^2^ = 26%). Mixed use carried an 80% increased risk (RR = 1.80, 95%CI 1.60–2.02, P = 0.001, *I*^2^ = 0%).

#### Subgroup analyses

The following subgroup analyses were was to explore the heterogeneity and found to explain the heterogeneity: duration of medication; case mix of patients (insomniac-only studies or not); and the type of study design (population based or non-population based studies).

## Discussion

This is the first meta-analysis to have compared the association between hypnotic medications and hip fracture, according the length of usage. We found an increase in the association between both BNZ and Z-drug use, and hip fracture. There appeared little difference in the findings between BNZ and Z-drugs. Our findings reinforce the evidence base that has highlighted the increased risk of hip fracture following BNZ or zopiclone use [27–29].

Importantly this study has outlined the potential dangers of all Z-drugs in relation to hip fractures, not solely zopiclone.

Furthermore, we found that the risk of fracture depended on the length of time people used their medication, since newly prescribed users of BNZ and Z-drugs were at the greatest risk of hip fracture, as previously postulated in the literature [36, 37]. This suggests the need for further supportive multifactorial intervention to prevent falls, these may include: education about the risks; strength and balance training; home assessment; vision assessment and referral; or physiotherapy [63]. These interventions should be considered at the beginning of hypnotic prescription to reduce the risk of fall and subsequent hip fracture.

### Biological mechanisms

BNZ and Z-drugs both induce sedation by enhancing the effect of the neurotransmitter GABA within the central nervous system. Consequently they may cause drowsiness, delayed reaction times and impair balance. We suggest that new users (up to 14 days) may be unaccustomed to potentiated levels of GABA prior to prescription. As such their risk of fall and subsequent hip fracture may be higher than patients with medium or long term hypnotic prescription.

NICE currently recommends that hypnotics should be prescribed to patients with severe insomnia, at the lowest dose for the shortest period of time [17]. However, Berry et al. 2013, Bakken et al. 2014, Chang et al. 2008 and Zint et al. 2010 suggested significant harm with even short-term prescriptions. Our study has highlighted the immediate risk of hip fracture amongst new BNZ or Z-drugs users, which is not addressed in the current NICE guidance [36, 37, 64]. This work raises debate of the risk-benefit of hypnotics and anxiolytics, and the need to explore the relative effectiveness of other (often non-pharmacological) approaches to these conditions.

### Strengths & limitations

There was a consistent direction of effect across all included studies and clear findings across all eighteen studies of an increased risk. No studies were included that directly compared BNZ versus Z-drugs for this outcome, so no direct comparison could be made between the two exposures. All studies included were non-randomised, and there was heterogeneity in some of the meta-analyses, so in these findings need to be taken with caution. The majority of studies measured dispensing or prescription data, and thus could not confirm patient adherence. Studies adjusted for a variety of covariates, and factors (S4 File), but none of the studies address non-registered drug use or concomitant alcohol. Many patients take BNZ and Z-drugs as and when needed, so the exposure of these drugs may vary throughout the studies. The cause of the fall may be in some cases due to other causes (e.g. the insomnia itself) rather than the medicine given to treat it.

### Recommendations for future research

Adequately powered RCTs exploring a head-to-head comparison of short acting BNZ v Z-drugs and risk of hip fracture would be useful to investigate which medicine (if any) has the better safety profile. Also adequately powered RCTs investigating interventions for newly prescribed users are needed to minimise the ‘new user’ effect that we have found, for example the use of a patient information sheet highlighting what expect, and what routine tasks to avoid, when newly prescribed BNZ or Z-drugs. In addition, research into alternative non-pharmacological interventions is needed to determine the best use of hypnotics within the wider context of clinical practice.

### Recommendations for future clinical practice

Patients require clearer information about the risks associated with hypnotics. Attention needs to be drawn to the increased risk of falling when prescribing BNZ and Z-drugs to new patients. Clinical guidance and education needed to reduce prescribers’ perceptions concerning the relative risk benefit of Z-drugs compared with BNZs. Finally, the length of hypnotic prescription needs to be carefully re-evaluated for each individual within the broader clinical context. Long term prescription carries well documented risks- dependence, falls and cognitive impairment. This study reinforces the need to carefully evaluate the indications for BNZ or Z drug prescription in older persons and to consider if multifactorial intervention might be necessary, as outlined in the NICE clinical guideline “Falls in older people: assessing risk and prevention “[63].

## Conclusions

This review indicated a similar risk profile of hip fracture for individuals receiving Z-drugs or receiving BNZs. We have highlighted the greatest risk appears to be in patients newly prescribed hypnotic/anxiolytic agents. Alternative non-drug options need to be considered for even short-term use (e.g. in treating short-term insomnia) in order to reduce the risk of fall and subsequent hip fracture, particularly in the older person.

## Supporting information

S1 File PRISMA checklist. (PDF)

S2 File Systematic review protocol for “Benzodiazepines, Z-drugs and the risk of hip fracture: A systematic review and meta-analysis”. (DOCX)

S3 File Appendix 1—Medline via Ovid search strategy. (DOCX)

S4 File Characteristics of included studies: (a)- Case control studies; (b)—Cohort studies. (DOCX)

S5 File Sensitivity analysis funnel-plot, showing the three subgroup. (DOCX)
