# Supplementary material for: Benzodiazepines, Z-drugs and the risk of hip fracture: A systematic review and meta-analysis
Source: PLoS One. 2017 Apr 27;12(4):e0174730. doi: 10.1371/journal.pone.0174730 (PMC5407557; doi:10.1371/journal.pone.0174730)
Supplement: S2 File — (DOCX) [file pone.0174730.s002.docx]

**Systematic Review Protocol for “Benzodiazepines, Z-drugs and the risk of hip fracture: A Systematic Review and Meta-Analysis”**

**Title**

The risk of hip fracture associated with BNZ or Z-drug use: A systematic manner and meta-analysis.

**Overall objective**

The overall objective of the meta-analysis is to compare the risk of hip fracture between Z-drugs and BNZ medications, and determine if this is attenuated over longer use.

**Research question**

What is the impact of Z-drugs, or Benzodiazepine (BNZ) use on the risk of fracture? And is the effect attenuated over time

**Objectives**

1. To determine whether Z-drugs have an effect on hip of fracture relative to length of usage.
2. To determine whether BNZ use has an effect on risk of hip fracture relative to length of usage.

**Preliminary literature review**

Medline via Ovid (EMBASE, Ovid, Psych INFO) was used to carry out a primary literature search. The Following combination of terms will be used in the search: (Hip Fractures OR Femoral Fractures OR Femoral Neck Fractures) AND (Benzodiazepines OR Diazepam OR Lorazepam OR Chlordiazepoxide OR Oxazepam OR Temazepam OR Nitrazepam OR Lorazepam OR Clobazam OR Clonazepam OR (Z-drug OR Zopiclone OR Zaleplon OR Zolpidem OR Hypnotic and Sedative) AND (Aged OR Elderly) AND (Review OR Meta-analysis). Thirty two titles and abstracts were screened resulting in 7 eligible studies.

Two Meta –analyses and 5 literature review of interest were found. All studies demonstrated the increased hip fracture risk associated with benzodiazepine use. Khong et al. 2012 conducted a literature review by which they demonstrated an increased fracture risk following benzodiazepine use. Grad et al. 2005 demonstrate the association between long-acting benzodiazepines and hip fracture in the community dwelling elderly. Both Huang et al. 2012 and Woolcot et al. 2009 carried out meta-analyses in which they demonstrated a significant association with benzodiazepines and hip fracture and also between hypnotics and sedatives and hip fracture. The most recent systematic review and meta-analysis found was conducted by Xing et al. (2014) which found that benzodiazepines carry a moderate and clinically significant hip fracture risk.

However, it must be noted that none of these studies explored whether there was a fracture risk associated with Z-drug usage. Alarmingly, Siriwardena et al. 2006 report that general practitioners believe Z-drugs are a safer and more effective alternative than BNZ with a similar safety profile. Secondly, current NICE guidelines recommend limiting benzodiazepine prescription to short-term use. Importantly only one of these studies, , a non-systematic review by Cumming et al 2003, distinguished between short term and long term usage of benzodiazepines with regards to hip fracture risk.

Indeed the scoping the literature has demonstrated a significant association between benzodiazepine use and hip fracture risk. However there still begs two important research questions:

1) What is the relative safety of Z-drugs as an alternative to benzodiazepines?

2) What is the relationship between hip fracture and length of benzodiazepine or Z-drug prescription?

There is a need for high quality systematic review and meta-analysis to deduce the association of Z-drugs with hip fracture and to determine the relationship between length of benzodiazepine or z-drug prescription and fracture risk.

**Research design and methodology**

The study is designed as a systematic review and meta-analysis.

Search strategy:

Peer-reviewed articles will be searched in the following electronic databases: Medline via Ovid (EMBASE, Ovid, Psych INFO) and Scopus. The Following combination of terms will be used in the search: (Hip Fractures OR Femoral Fractures OR Femoral Neck Fractures) AND (Benzodiazepines OR Diazepam OR Lorazepam OR Chlordiazepoxide OR Oxazepam OR Temazepam OR Nitrazepam OR Lorazepam OR Clobazam OR Clonazepam OR (Z-drug OR Zopiclone OR Zaleplon OR Zolpidem OR Hypnotic and Sedative) AND (Aged OR Elderly) AND published 2005-2015.

Inclusion criteria:

Studies were included if all of the following criteria applied (i) designed as a randomised controlled trial, cohort or case-control study (ii) reported outcome was hip fracture (ICD-10: S72) or fragility fracture (within which outcome ≥70% of fractures were hip fractures) (iv) included patients were prescribed either BNZ or Z-drug, or were matched as a non-exposed control population (v) the study population were aged at least 50 years old and with a mean age over 65. All searches to identify suitable studies will be duplicated by an independent researcher, and disagreements found will be discussed. All data extracted will also be independently carried out and any disagreements resolved through discussion.

Outcome and measure of treatment effect:

The proportion of individuals reported with a hip fracture.

Exclusion criteria:

Clinically, clonazepam is frequently used as an anti-epileptic rather than as a hypnotic and was therefore excluded.

**Data analysis**

Quality of individual studies will be assessed using relevant Newcastle-Ottawa Scale and then the overall quality will be rated good, fair or poor according to three-grade system. Case control studies will be assessed according the following domains: (i) Selection (ii) Comparability and (iii) Exposure. Cohort studies will be assessed according to the domains: (i) Selection (ii) Comparability (iii) Outcome.

Subgroup analysis: Following subgroup analyses will be performed.

1. Length of usage

2. Non-population based and population based studies

Data synthesis: Only clinical similar studies with the same outcome in the same context will be considered for pooling into a meta-analysis. Pooling will apply a random effect model and present a relative risk summary with 95% CI and associated p-value. Any heterogeneity found will be explored

Heterogeneity assessing: Heterogeneity will first be visually assessed through graphical presentation of forest plot. Then, it will be statistically assessed by

Cochran’s Q-test with a *p*-value of 0.1 for statistical significance and be quantified by *I^2^ statistic.*

Publication bias: Funnel plot will be used to visually inspect publication bias. If at least 10 studies are included in the meta-analysis, a modified version of the linear method will be used to objectively assess publication bias.

Sensitivity analysis: Sensitivity analysis will be performed by restricting the meta-analysis to studies with good quality or fair quality.

Rating the strength of evidence: Following domains will be assessed: study limitations, directness, consistency, precision and reporting bias.
