# Supplementary material for: Benzodiazepines, Z-drugs and the risk of hip fracture: A systematic review and meta-analysis
Source: PLoS One. 2017 Apr 27;12(4):e0174730. doi: 10.1371/journal.pone.0174730 (PMC5407557; doi:10.1371/journal.pone.0174730)
Supplement: S3 File — (DOCX) [file pone.0174730.s003.docx]

Medline via Ovid Search Strategy

Hip Fracture + (BNZs + Z-drugs) + Elderly

1. exp Hip Fractures/

2. (hip* adj3 fracture*).mp. [mp=title, abstract, original title, name of substance word, subject heading word, keyword heading word, protocol supplementary concept word, rare disease supplementary concept word, unique identifier]

3. exp Femoral Fractures/ or exp Femoral Neck Fractures/

4. (femoral* adj neck* adj3 fracture*).mp. [mp=title, abstract, original title, name of substance word, subject heading word, keyword heading word, protocol supplementary concept word, rare disease supplementary concept word, unique identifier]

5. 1 or 2 or 3 or 4

6. exp Benzodiazepines/

7. benzodiazepine*.mp. [mp=title, abstract, original title, name of substance word, subject heading word, keyword heading word, protocol supplementary concept word, rare disease supplementary concept word, unique identifier]

8. exp Diazepam/

9. diazepam*.mp. [mp=title, abstract, original title, name of substance word, subject heading word, keyword heading word, protocol supplementary concept word, rare disease supplementary concept word, unique identifier]

10. exp Lorazepam/

11. lorazepam*.mp. [mp=title, abstract, original title, name of substance word, subject heading word, keyword heading word, protocol supplementary concept word, rare disease supplementary concept word, unique identifier]

12. exp Chlordiazepoxide/

13. chlordiazepoxide*.mp. [mp=title, abstract, original title, name of substance word, subject heading word, keyword heading word, protocol supplementary concept word, rare disease supplementary concept word, unique identifier]

14. exp Oxazepam/

15. oxazepam*.mp. [mp=title, abstract, original title, name of substance word, subject heading word, keyword heading word, protocol supplementary concept word, rare disease supplementary concept word, unique identifier]

16. exp Temazepam/

17. temazepam*.mp. [mp=title, abstract, original title, name of substance word, subject heading word, keyword heading word, protocol supplementary concept word, rare disease supplementary concept word, unique identifier]

18. exp Nitrazepam/

19. nitrazepam*.mp. [mp=title, abstract, original title, name of substance word, subject heading word, keyword heading word, protocol supplementary concept word, rare disease supplementary concept word, unique identifier]

20. loprazolam.mp.

21. exp Lorazepam/ or lormetazepam.mp.

22. clobazam.mp.

23. exp Clonazepam/

24. clonazepam*.mp. [mp=title, abstract, original title, name of substance word, subject heading word, keyword heading word, protocol supplementary concept word, rare disease supplementary concept word, unique identifier]

25. 6 or 7 or 8 or 9 or 10 or 11 or 12 or 13 or 14 or 15 or 16 or 17 or 18 or 19 or 20 or 21 or 22 or 23 or 24

26. exp Aged/

27. aged*.mp. [mp=title, abstract, original title, name of substance word, subject heading word, keyword heading word, protocol supplementary concept word, rare disease supplementary concept word, unique identifier]

28. elderly*.mp. [mp=title, abstract, original title, name of substance word, subject heading word, keyword heading word, protocol supplementary concept word, rare disease supplementary concept word, unique identifier]

29. 26 or 27 or 28

30. exp "Hypnotics and Sedatives"/

31. Z drug*.mp. [mp=title, abstract, original title, name of substance word, subject heading word, keyword heading word, protocol supplementary concept word, rare disease supplementary concept word, unique identifier]

32. zaleplon*.mp. [mp=title, abstract, original title, name of substance word, subject heading word, keyword heading word, protocol supplementary concept word, rare disease supplementary concept word, unique identifier]

33. zolpidem*.mp. [mp=title, abstract, original title, name of substance word, subject heading word, keyword heading word, protocol supplementary concept word, rare disease supplementary concept word, unique identifier]

34. zopiclone*.mp. [mp=title, abstract, original title, name of substance word, subject heading word, keyword heading word, protocol supplementary concept word, rare disease supplementary concept word, unique identifier]

35. 30 or 31 or 32 or 33 or 34

36. 25 or 35

37. 5 and 29 and 36

38. limit 37 to (english language and yr="2005 -Current")

49 Results
