# Supplementary material for: Benzodiazepines, Z-drugs and the risk of hip fracture: A systematic review and meta-analysis
Source: PLoS One. 2017 Apr 27;12(4):e0174730. doi: 10.1371/journal.pone.0174730 (PMC5407557; doi:10.1371/journal.pone.0174730)
Supplement: S4 File — (DOCX) [file pone.0174730.s004.docx]

| **Study**  **Supplementary Table 1 (a)- Case Control Studies** | **Location** | **Environment** | **Design** | **Drug** | **Fracture** | **Age (yrs)** | **Mean age (yrs)** | **Sample size** | **Cases** | **Controls** | **Length of use (short/ long/ mixed term)** | **OR or RR** | **CI (95%)** | **Adjusted for Confounders** | **Dosage adjusted for (yes/no)** |
| --- | --- | --- | --- | --- | --- | --- | --- | --- | --- | --- | --- | --- | --- | --- | --- |
| Berry 2013 | US | Nursing Homes | Case-crossover | Z-drugs | Hip | ≥50 | 81 | 15528 | 1715 | Self-controlled | 0-15days  **(Short)** | 2.20 | 1.76-2.74 | Participants were compared with themselves | No |
|  |  |  |  |  |  |  |  |  |  |  | 0-30days  **(Medium)** | 1.90 | 1.60-2.26 |  |  |
| Bolton 2008 | Canada | Population Data Base | Case-crossover | BNZ | Hip wrist or vertebral | ≥50 | Not stated | 63081 | 15792 fracture patients; 3066 used BNZ | 47289 non-fracture patients; 7727 used BNZ | 0-120days  **(Mixed)** | 1.10 | 1.04-1.16 | Frailty | Yes  In tertiles  i)low  ii)moderate  iii)high |
| Chang 2008 | Taiwan | National Health Insurance Database | Nested CC | BNZ | Hip | ≥65 | 78.2 | 1431 | 217 fracture patients; 57 BNZ users | 1214 non-fracture patients; 185 used BNZ | 0-30days  **(Mixed)** | 5.60 | 2.7-11.8 | Antidepressant exposure, no. of outpatient visits, comorbidity | In tertiles of Diazepam Equivalent Dose:  i)low 0.1-3mg  ii)moderate 3.1-6mg  iii)high >6mg |
|  |  |  |  |  |  |  |  |  |  |  | 31-90days  **(Long)** | 1.40 | 0.70-2.8 |  |  |
|  |  |  |  |  |  |  |  |  |  |  | >90 days  **(Long)** | 1.30 | 0.80-2.10 |  |  |
|  |  |  |  |  |  |  |  |  |  |  | Mixed | 1.70 | 1.2-2.50 |  |  |
| Coutinho 2008 | Brazil | Hospitals | CC | BNZ | Femur, Forearm, Vertebral | >60 | 75.4 | 500 | 250 fracture cases | 250 | Taken  0-24hrs before index date  **(Mixed)** | 2.22 | 1.07-4.58 | Socio-demographic impacts on health, BMI and cognitive impairment | No |
| Golden 2010 | US | Medicare Health Insurance Database | CC | LABNZ | Hip, radius & ulna, ribs, pelvis, skull, multiple | ≥65 | 79.88 | 16328 | 376 | 168 | 0-3months  **(Mixed)** | 1.90 | 1.49-2.43 | Age, antidepressants, antipsychotics, non-BNZ sedatives, tension headache, fibromyalgia, urinary incontinence, back disorders | No |
|  |  |  |  | SABNZ |  |  |  |  | 989 | 624 | 0-3months  **(Mixed)** | 1.33 | 1.15-1.55 |  |  |
| Kang 2012 | S. Korea | Health Insurance Database | Case-crossover | Zolpidem | Femur, Skull, Vertebral, upper limb, hand, lower limb | ≥65 | Not stated | 1758 | 364 | 1394 | 0-180days  **(Mixed)** | 1.72 | 1.37-2.16 | Co-medications: AEDs, antipsychotics, analgesics, antidepressants (anticholinergics), calcium channel blockers | No |
|  |  |  |  | BNZ |  |  |  |  |  |  |  | 1 | 0.83-1.12 |  |  |
| Perreault 2008 | Canada | Quebec Health Databases | Nested CC | BNZ | Hip, Colles, Vertebral, Rib, Humerus, Hand, Ankle, Tarsus or metatarsal | ≥70 | 75.7 | 20064 | 1824 female fracture patients; 91 used BNZ | 18240 female non-fracture patients; 876 used BNZ | 0-1year  **(Mixed)** | 1.26 | 1.11-1.42 | City/rural living, total number of physicians seen per year, diabetes, time since diagnosis or last fracture, co-medications: LABNZ, antidepressants, narcotics and antiepileptic drugs(AEDs) | No |
| Pierfitte 2001 | France | A&E | CC | BNZ | Hip | ≥65 | Not stated | 1062 | 245 fracture patients; 83 used BNZ | 817 non-hip fracture patients; 294 used BNZ | **(Mixed)** | 0.98 | 0.72-1.34 | Age, sex, height, BMI, living environment, date and hospital of admission | No |
| Wang 2001 | US | New Jersey Medicaid | CC | Zolpidem | Hip | ≥65 | 82 | 6110 | 1222 hip fracture patients | 4888 non-hip fracture patients | 0-6months  **(Mixed)** | 1.95 | 1.09-3.51 | Age, comorbidities, gender, race, previous psychoactive medication exposure, previous care home or hospital stay | No |
|  |  |  |  | BNZ |  |  |  |  | 1222 hip fracture patients; 198 used BNZ | 4888 non-hip fracture patients; 523 used BNZ |  | 1.46 | 1.21-1.76 |  |  |
| Zint 2010 | US | Medicare | Nested CC | BNZ | Hip | ≥65 | 84.3 cases 79.5  controls | 103188 | 17198 fracture cases; 2840 used BNZ | 85990 non-fracture patients; 11410 used BNZ | 0-14days  **(Short)** | 2.05 | 1.52-2.77 | Co-medications, comorbidities, length of hospital/care home stay, socio-demographic status. | Yes  In dose tertiles;  i)Low <0.5 DDD  ii)Moderate 0.5-1  iii)High >1 |
|  |  |  |  |  |  |  |  |  |  |  | 15-30days  **(Medium)** | 1.42 | 1.03-1.96 |  |  |
|  |  |  |  |  |  |  |  |  |  |  | 31-60days  **(Long)** | 1.34 | 1.02-1.77 |  |  |
|  |  |  |  | Zolpidem |  |  |  |  |  |  | 0-14days before index date  **(Mixed)** | 1.26 | 1.11-1.44 |  |  |

| **Study**  **Supplementary Table 1 (b)-** **Cohort Studies** | **Location** | **Environment** | **Design** | **Drug** | **Fracture** | **Age (yrs)** | **Mean age**  **(yrs) (SD)** | **Sample size** | **Length of use (new user or short/ long/ mixed term)** | **OR or RR** | **CI (95%)** | **Study Period** | **Adjusted for Confounders** | **Dosage Adjusted for (yes/no)** |
| --- | --- | --- | --- | --- | --- | --- | --- | --- | --- | --- | --- | --- | --- | --- |
| Bakken 2014 | Norway | Population based | Cohort (prospective) | SABNZ | Hip | ≥59 | 72.8  (8.9) | 906422: 2009 cases of Hip Fracture & BNZ users | Exposed person days  **(Mixed)** | 1.5 | 1.4-1.6 | 2004-2010 | Sex, birth year, time elapsed since fracture | Yes |
|  |  |  |  | LABNZ |  |  |  |  | Exposed Person Days  **(Mixed)** | 1.2 | 1.2-1.3 |  |  |  |
|  |  |  |  | Z-drugs (day) |  |  |  |  | 0-14days  **(Short)** | 1.2 | 1.1-1.4 |  |  |  |
|  |  |  |  |  |  |  |  |  | Exposed person days **(Mixed)** | 1.1 | 1.1-1.2 |  |  |  |
|  |  |  |  | Z-drugs (night) |  |  |  |  | 0-14days  **(Short)** | 1.4 | 1.2-1.5 |  |  |  |
|  |  |  |  |  |  |  |  |  | Exposed person days **(Mixed)** | 1.3 | 1.2-1.4 |  |  |  |
| Chan 2010 | Taiwan | Health Insurance Database | Cohort (retrospective) | BNZ | Hip | ≥65 | Not stated | 62023 patients | 7-180 days  **(Mixed)** | 1.57 | 1.32-2.3 | 2000-2003; yearly follow up | Age, sex, co-morbidity, co-medications | Inappropriate doses studied (Beers Criteria) |
| Cummings 1995 | USA | Postal questionnaire | Cohort | LABNZ | Hip | ≥65 | 72(5) | 9516 white women; 875 fracture patients; 192 used BNZ | 0-1year **(Mixed)** | 1.6 | 1.1-2.4 | 1986-1988; 4.1year follow up | Age, co-medications, bone mineral density | No |
|  |  |  |  | SABNZ |  |  |  |  |  | 1.2 | 0.8-2.1 |  |  |  |
| Ensrud 2003 | USA | Community Based | Cohort (prospective) | BNZ | Hip | ≥65 | 77(4.7) | 8127 white women; 626 used BNZ | 0-14days before index date  **(Mixed)** | 1.2 | 0.7-2.0 | 1992-1999; 4.8year follow up | Age, BMD, confounding by indication. Single blinded | No |
| Finkle 2011 | USA | Community based | Cohort (retrospective) | Lorazepam | Hip | ≥65 | 53.8 | 43343 patients | 0-15days  **(Short)** | 2.63 | 1.83-3.80 | 1999-2009 | Age, co-morbidities, co-medications | No |
|  |  |  |  |  |  |  |  |  | 16-30days  **(Medium)** | 1.48 | 0.93-2.37 |  |  |  |
|  |  |  |  |  |  |  |  |  | 31-60days  **(Long)** | 1.02 | 0.71-1.47 |  |  |  |
|  |  |  |  |  |  |  |  |  | 0-90days **(Mixed)** | 1.53 | 1.23-1.91 |  |  |  |
|  |  |  |  | Diazepam |  |  | 53.4 | 150858 patients | 0-15days  **(Short)** | 3.94 | 2.11-7.35 |  |  |  |
|  |  |  |  |  |  |  |  |  | 16-30days  **(Medium)** | 1.52 | 0.59-3.92 |  |  |  |
|  |  |  |  |  |  |  |  |  | 31-60days  **(Long)** | 1.25 | 0.59-2.65 |  |  |  |
|  |  |  |  |  |  |  |  |  | 0-90days **(Mixed)** | 1.97 | 1.22-3.18 |  |  |  |
|  |  |  |  | Zolpidem |  |  | 49.1 | 93618 patients | 0-15days  **(Short)** | 3.28 | 1.74-6.19 |  |  |  |
|  |  |  |  |  |  |  |  |  | 16-30days  **(Medium)** | 3.58 | 1.90-6.75 |  |  |  |
|  |  |  |  |  |  |  |  |  | 31-60days  **(Long)** | 2.60 | 1.51-4.46 |  |  |  |
|  |  |  |  |  |  |  |  |  | 0-90days **(Mixed)** | 2.55 | 1.78-3.65 |  |  |  |
| Guo 1998 | Sweden | Community | Cohort (prospective) 1987-1993 | BNZ | Hip | ≥75 | Unknown | 1810; 209 used BNZ; 134 hip fractures | 0-14 before index date  **(Mixed)** | 1.41 | 0.90-2.9 | 1987-1993 | Age, sex, NH residence, education, history of stroke or tumour, functional, visual and mental capacity, | No |
| Kragh 2011 | Sweden | 2 National Databases | Cohort | BNZ | Hip | >60 | 83.6 | 2043 hip fracture patients | 0-6months  **(Mixed)** | 1.72 | 1.3-2.27 | 2006;  6 months studied | Age | No |
| Thorell 2014 | Sweden | Register-based | Cohort (prospective) | Anxiolytic | Hip | ≥75 | Unknown | 38407 patients; 795 hip fracture and 37612 non-fracture patients | 0-1year  **(Mixed)** | 1.31 | 1.11-1.54 | 2006-2007 | Age, gender, multi-morbidity level | No |
|  |  |  |  | Hypnotic |  |  |  |  |  | 1.31 | 1.13-1.52 |  |  |  |
| Wagner 2004 | US | New Jersey Medicaid claims | Cohort (retrospective) | BNZ | Hip | ≥65 | Not stated | 125203: 30696 used BNZ, 2312 hip fractures | 0-15days  **(Short)** | 2.05 | 1.28-3.28 | 1987-1990 enrolment; 42 months follow up | Age, gender, ethnicity, NH residence, hospital admission in past months, psychoactive co-medications, epilepsy, dementia | No |
|  |  |  |  |  |  |  |  |  | 16-30days  **(Medium)** | 1.88 | 1.15-3.07 |  |  |  |
|  |  |  |  |  |  |  |  |  | 31-210 days  **(Long)** | 1.18 | 1.03-3.07 |  |  |  |
|  |  |  |  |  |  |  |  |  | 0-210 days **(Mixed)** | 1.24 | 1.06-1.44 |  |  |  |
