# Supplementary figures and images for: Benzodiazepines, Z-drugs and the risk of hip fracture: A systematic review and meta-analysis
Source: PLoS One. 2017 Apr 27;12(4):e0174730. doi: 10.1371/journal.pone.0174730 (PMC5407557; doi:10.1371/journal.pone.0174730)

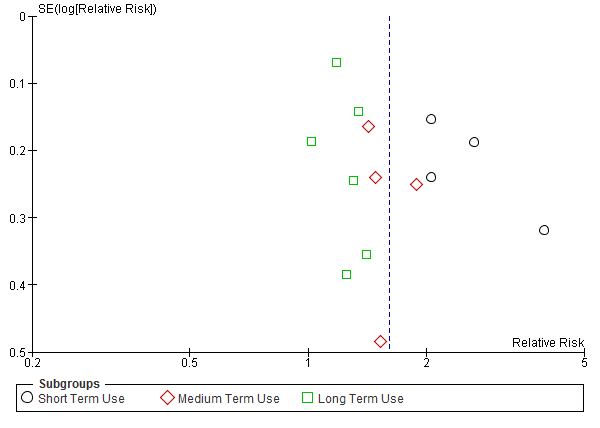
Supplementary Figure 1 – Sensitivity analysis funnel plot, showing the three subgroup

Supplement: S5 File — (DOCX) [file pone.0174730.s005.docx]
